# Trauma networks: present and future challenges

**DOI:** 10.1186/1741-7015-9-121

**Published:** 2011-11-11

**Authors:** Nikolaos K Kanakaris, Peter V Giannoudis

**Affiliations:** 1 Academic Department of Trauma and Orthopaedics, Leeds General Infirmary, Clarendon Wing, Level A, Great George Street, LS13EX, Leeds, UK

## Abstract

In England, trauma is the leading cause of death across all age groups, with over 16,000 deaths per year. Major trauma implies the presence of multiple, serious injuries that could result in death or serious disability. Successive reports have documented the fact that the current *ad hoc *unstructured management of this patient group is associated with considerable avoidable death and disability. The reform of trauma care in England, especially of the severely injured patient, has already begun. Strong clinical leadership is embraced as the way forward. The present article summarises the steps that have been made over the last decade that led to the recent decision to move towards a long anticipated restructure of the National Health Service (NHS) trauma services with the introduction of Regional Trauma Networks (RTNs). While, for the first time, a genuine political will and support exists, the changes required to maintain the momentum for the implementation of the RTNs needs to be marshalled against arguments, myths and perceptions from the past. Such an approach may reverse the disinterest attitude of many, and will gradually evolve into a cultural shift of the public, clinicians and policymakers in the fullness of time.

## Background

Trauma consists of a wide spectrum of clinical conditions that are initiated in the immediate aftermath following injury. In England, trauma is the leading cause of death across all age groups, with over 16,000 deaths per year. It is one of the few diseasecategories in which mortality is increasing [1-5].

The term 'major trauma' is used to describe a serious clinical state of injured patients, and often implies the simultaneous presence of multiple injuries to the same or different body regions and systems [6]. For the purposes of a healthcare system, it also includes any injury so complex that it exceeds the capabilities or expertise of the receiving health unit [7]. From the health economic aspect point of view, 'multiple trauma' or 'polytrauma' is represented by Healthcare Resource Groups (HRG)-4 codes (subchapter VA; 8 different codes), whereas from a clinical research point of view it is defined as an injury severity score (ISS) of more than 16 in the presence of more than 1 injury [8,9]. Irrespective of the differing definitions, limitations and disparity, major trauma is proven to affect 15% of all injured patients; less than 2 per 1,000 emergency department admissions, around 30 cases per 100,000 of population per year [7]. It is the sixth highest overall leading cause of death and fifth highest cause of disability in all age groups across the globe [10]. In England there are at least 20,000 such cases annually, which are managed in 193 different hospitals, with an estimated cost of £0.3 to £0.4 billion per year [6].

Successive reports (Table 1) have documented the fact that the current *ad hoc *unstructured management of this patient group is associated with considerable avoidable death and disability. The high acuity and need for trauma readiness around the clock, the reality of delayed secondary transfers, and the justified severe criticism of the currently offered care and waste of resources all set the scene for an urgently needed reformat of trauma services in the England [6,11-38].

**Table 1:** Calendar of related past events

Source, year	Statement	Recommendation
Osmond-Clark H, 1961-1975 [11-13]	'Casualty department' staffed by casualty officers, usually senior house officers (SHOs) who had been qualified for 1 or 2 years. Senior cover was negligible.	Tripartite scheme of peripheral casualty units, DGH accident centres and a regional major injury unit serving a population of 1 to 2 million

Cales RH and Trunkey DD, 1985 [14]	Effect of a regional trauma system in reducing trauma mortality (USA)	Use of 'preventable trauma deaths' as an evaluation tool of effectiveness

RCSE report, 1988 [15]	Great discrepancy and deficiencies in existing emergency services. Serious deficiencies in the management of seriously injured patients.	Establishment of trauma centres. Introduction of ATLS in the UK. Three pilot trauma centres in the UK (Royal London Hospital, the John Radcliffe Hospital in Oxford and North-Staffordshire Hospital in Stoke).

Anderson ID, 1988 [16]	Preventable trauma deaths over 33% in England and Wales	Need for changes in trauma-related services

BOA report, 1989 [17]	Too many small units and too few consultants with a special interest in trauma care. Considerable resource implications of trauma centres (American model).	Services should be concentrated. Need for expansion of consultant numbers.

Redmond AD, 1991 [18]	First trauma centre in the UK (North Staffordshire Hospital Centre). Cost effectiveness and appropriateness evaluation.	Use of pilot programmes to template needed changes

NAO report, 1992 [19]	Deficiencies in NHS Accident and Emergency (A&E) Departments in England	Information and Actions are required. Early and continuing improvements are needed. How trauma audit should be taken forward.

BOA report, 1992 [20]	Review of 263 hospitals in the UK identified deficiencies in staff and equipment. Trauma services had not kept pace with technical advances. Many units were too small to sustain an adequate standard of care.	Need for Regional Trauma Centre with its multidisciplinary arrangements. Set standards for the facilities required in a DGH. Rapid transfer to suitable hospital. Direct involvement of senior clinicians.

MTOS Study, Yates DW *et al*., 1992 [21]	Initial resuscitation by junior staff in more than 50% of the cases. Delays in providing experienced staff and timely operations. Mortality varies inexplicably between hospitals. Significantly higher mortality rate for blunt trauma than in the US.	Reformation of Trauma Services. Early senior staff involvement.

UK-TARN formation, 1993 [22]	Creation of Data Collection Network related to trauma	Recording performance and allowing the rationalisation of implemented changes to the trauma services

Rowley DI, 1993 [23]	Lack of A&E consultants (21%). Lack of vital trauma associated specialties (80%). Lack of intensive care facilities (6%).	Reformation of trauma services. Early senior staff involvement.

Nicholl J and Turner J, 1997 [24]	Evaluation of pilot trauma centre and regional network showed modest reductions in mortality	Greater integration along the entire trauma care pathway is the priority

BOA report, 1997 [25]	Lower quality of care in comparison to countries such as Germany, Switzerland and the USA	An integrated network approach to treating trauma patients. Integrated approach based upon a hub and spoke model.

BOA and RCSE joint report, 2000 [26]	Current system does not assess the quality of life of those that survive. Only 50% of Trusts subscribe to TARN.	Need for nationally coordinated standards of care. Need for systematic audit. Need for the development of outcome measures. Need for geographical trauma systems. Need for a strategy for rehabilitation.

Lecky FE, *et al*., 2002 [27]	Lack of significant improvement in case fatality reduction between 1994 and 2000 according to the UK-TARN data	

NAO report, 2004 [28]	Trauma audit has been improved through the establishment of TARN. Still scope for this work to be developed at a regional level	Further expansion of the national TARN network. Development of Regional services.

NCEPOD report, 2007 [6]	Deficiencies in both the organisational and clinical aspects of trauma care. Organisation of prehospital care, trauma team response, seniority of staff involvement and immediate in-hospital care was found to be deficient. Less than good care for 60% of reviewed major trauma patients.	Need for designated Level 1 trauma centres. Ensure that a trauma team is available 24/7. A consultant must be the team leader for the management of the severely injured patient.

Lord Darzi's London report, 2007 [29]	Review of London's healthcare identified stark of inequalities in health outcomes and the quality and safety of patient care not as good as it could, and should, be	A trauma system should be put into operation within London. Integration of hospital and prehospital care. Bypass protocols need to be traduced taking the most seriously ill directly to trauma centres.

Lord Darzi's report, 2008 [30]	Review of the NHS identified compelling arguments for saving lives by creating specialised centres for major trauma. SHA are asked to begin considering major trauma services.	Create specialised centres for major trauma. Development of regional plans from strategic health authorities

RCSE report, 2009 [31]	Without regionalisation, trauma mortality and morbidity in the UK will remain unacceptably high. The likelihood of dying from injuries has remained static since 1994 despite improvements in trauma care, education and training.	Individual SHAs need to interpret the guidance to meet their own needs. There is no 'fit-all' scenario. Further development is urgently needed regarding areas as paediatric trauma care, burns care and rehabilitation services.

Professor Keith Willett, 2009	Department of Health appoints a National Clinical Director for Trauma care for the first time.	National leadership for the implementation of regional trauma networks in England. Commissioning, audit, modelling, metrics, standards, critical care capacity, interventional radiology, rehabilitation, behavioural change, workforce, and training needs.

Dr Fiona Moore, 2009	Healthcare for London appoints London's first Trauma Director	Responsible for leading the implementation of new specialist trauma networks across the capital

NAO report, 2010 [19]	Significant data gaps and a lack of formalised systems remain. Still 59% of hospitals delivering trauma care participate in UK TARN. Major trauma care cannot be delivered cost effectively by all hospitals. Only one hospital has consultant lead services 24/7. A total of 64% of major trauma patients do not receive specialist care. Unacceptable variations in mortality rates, depending on where and when a person receives treatment. Lack of adequate data on level of rehabilitation services. Current funding arrangements do not reflect the actual trauma costs.	SHA to develop regional trauma networks. Designation of hospitals suitable to receive major trauma cases. The DoH to review the financial levers of delivery of major trauma care.

London Network, 2010 [32]	Initiation of the first comprehensive Trauma Network of the UK. Major Trauma Centres: The Royal London Hospital (Whitechapel), St George's Hospital (Tooting), King's College Hospital (Denmark Hill) and St Mary's Hospital (Paddington).	Act also as a template for the development of the Regional Trauma Networks across the UK

Davenport DA, *et al*., 2010 [33]	The effect of the reform of trauma services at RLH and the introduction of a MDT trauma service in 2003 was identified to have reduced preventable deaths from 9% to 2%, and secondary transfer mortality by 53% versus the national average. Implementation of a specialist trauma service and performance improvement programme is associated with rapid reductions in mortality for the severely injured.	Future national major trauma centres should be specialist hospitals, not simply hospitals with specialties

East Midlands Network, 2011	Nottingham to be the first major trauma centre to start functioning outside London	Act also as a template for the development of the Regional Trauma Networks across the UK

BOA = British Orthopaedic, Association, DGH = District General Hospital, DoH = Department of Health, MDT = multidisciplinary team, MTOS = Major Trauma Outcome Study, NAO = National Audit Office, NCEPOD = National Confidential Enquiry into Patient Outcome and Death; NHS = National Health Service; RCSE = Royal College of Surgeons of England; RLH = Royal London Hospital; SHA = Strategic Health Authorities; TARN = Trauma Audit and Research Network.

The crude numbers representing the huge burden of trauma, the increasing public awareness of the issue, and the documented underperformance of the existing UK trauma services, eventually led to the recent decision for a long anticipated restructure of the National Health Service (NHS) trauma services. The present article aims to summarise the steps that have been taken over the last decade, culminating in the formation of Regional Trauma Networks (RTNs) nationally, as well as highlighting foreseeable future challenges.

### RTN rationale

The numerous official reports, mainly at a national level [6,11-13,15,19,20,25,26,28-31,39,40], and the accumulation of significant international experience [41-50] advocating organised networks of trauma care covering defined geographic regions, led to the recent evolution and maturation of decision making towards the formation of such a system in the UK. This challenging task was assigned by the Department of Health (DoH) to the Strategic Health Authorities (SHAs) in 2009 following the review of healthcare by the then Health Minister Lord Darzi [30]. The London and East Midlands areas have already gone a long way to completing their operational plan and will be used as pilots for the rest of the SHAs. The planning and designing of all networks by 2010-2011, and their implementation throughout the 2011-2012 financial year, was the original plan of the DoH and steps are rapidly being taken by all stakeholders to meet these deadlines.

The 'ideal' RTN is a complex all-inclusive system of trauma-related services, from prehospital care through to acute care and rehabilitation. It affects and is affected by numerous patient and healthcare-related parameters, as well as legislation and finances [51]. The goal is to match the utilised resources of the provider to the needs of the injured patients at the appropriate facility in a timely manner, achieving optimal management from the initial recognition of the injury to the return of the patient to the community.

A wholesale introduction of an American type of system does not appear suitable for the UK due to the presence of many densely populated areas, shorter transportation distances, and the already existing infrastructure and role of district general hospitals [24,26,31,35,37,39]. Nevertheless, the description of the fundamental components of a RTN made by the Committee on Trauma of the American College of Surgeons fully applies to the recently introduced UK model. They consist of: leadership (at all levels of trauma care delivery), trauma care facilities (major trauma centres, trauma units, transport services, rehabilitation units), human resources (planning and development, administrative and clinical teamwork), education-prevention-public awareness (information on the trauma system, communication pathways with primary care and the public), triage-transport means, interhospital transfers, communication network (at all levels of the trauma system), rehabilitation, and data collection and quality assurance monitoring [41].

The appointment for the first time of a National Clinical Director in 2009, and the subsequent appointment of London's Trauma Director, reflect the importance given to inspired and clinically experienced leadership at the highest level of planning, decision making and coordination. It signifies the fact that the reformation of trauma in the UK is finally being taken seriously. The role of leadership transects all through the pyramid of the introduced trauma system, with the assignment of regional and local trauma leaders with administrative and clinical representation at all levels.

Trauma care will be provided within the network, structured in different tiers of applied resources, ranging between trauma units and major trauma centres, based on each patient's need. The assignment of roles and subsequent accreditation and revalidation of trauma hospitals to the different tiers has been initiated by each SHA and local clinical advisory boards. One or more major trauma centres (MTCs) will be identified in each region by the SHA according to specific criteria (Table 2). The Royal College of Surgeons of England [52] recommended the allocation of one MTC for every 3-4 million inhabitants based on particular geographic and population characteristics as well as comprehensive evidence on the correlation of outcome to the trauma centre's volume [53,54], making a total number of 12-16 across England.

**Table 2:** Essential characteristics of hospitals delivering trauma care in the Regional Trauma Network (RTN) era

Characteristic	Types of Institutions offering care to injured patients	
	
	Major trauma centre (MTC)	Trauma unit (TU)
Total no. for England	12-16	80-100

Administration/governance:		

Commitment from executive team administration and senior clinical staff to deliver such services	100%	100%

Clinical director for trauma, programme manager and data manager	100%	100%

Full dataset submission to TARN	100%	100%

Commitment to RTN, trauma governance, continuous improvement	100%	100%

Critically injured patients (complex or major trauma)	>250 per year	Only for few cases when the bypass protocols are not followed (patient in extremis, accident in the premises of TU)

Accident and emergency (A&E): early resuscitation:		

A&E availability	24/7	24/7

Resuscitation trauma team leader	Consultant 24/7 resident	Experienced middle grade 24/7 resident; consultant on call^a^

Fully staffed with specialist nursing and allied health professionals	24/7	24/7

Band 7 nurse: specialist ED trauma nursing	24/7	Variable

Activation protocols: established prealert pathways	24/7	24/7

Trauma service:		

Ongoing patient care post initial resuscitation	Consultant on call^a^	Consultant on call^a^

Admission under a named consultant	Consultant on call^a^	Consultant on call^a^

Consultant-led service for ongoing coordination of the care of major trauma patients	Consultant on call^a^	Consultant on call^a^

Trauma nurse coordinator	Yes	Variable

Radiology: (X-ray/FAST-CT)	Reporting within 24 h	Reporting within 24 h

Available high specification CT scanner	24/7	24/7^a^

Staffed laboratory services	24/7	24/7

Blood bank	24/7	24/7

Massive transfusion protocol	24/7	24/7

Trauma and orthopaedic	Middle grade resident, consultant on call^a^, pelvic reconstruction service, complex limb reconstruction service	Middle grade resident, consultant on call^a^

General surgery	Middle grade resident, consultant on call^a^	Middle grade resident, consultant on call^a^

Neurosurgery	Middle grade resident, consultant on call^a^	Variable

Spinal surgery	Middle grade on call^a^, consultant on call^a^	Variable

Vascular surgery	Middle grade resident, consultant on call^a^	Middle grade resident, consultant on call^a^

Anaesthetist	Middle grade resident, consultant on call^a^; ODP support within resuscitation	Middle grade resident, consultant on call^a^

Plastic surgery	Middle grade on call^a^, consultant on call^a^	Variable

Urology	Middle grade on call^a^, consultant on call^a^	Variable

Cardio/thoracic surgery	Middle grade on call^a^, consultant on call^a^	Variable

Maxillofacial/head and neck	Middle grade on call^a^, consultant on call^a^	Variable

Interventional radiology	Angio suite available 24/7^a^, consultant on call^a^	Variable

Dedicated trauma theatres, operating lists	Immediately available, ability for second if overwhelmed	Immediately available, ability for second if overwhelmed

Intensive care facilities	24/7	24/7

Critical care	Middle grade resident, consultant on call^a^	Middle grade resident, consultant on call^a^

Miscellaneous:		

Education-prevention-research programmes	100%	Variable

Clinical governance programmes	100%	100%

Interhospital transfer protocols	100%	100%

Trauma rehabilitation programmes	100%	100%

^a^Available in 30 min.

CT = computed tomography; ODP = operating department practitioner.

Each MTC will be collaborating with a number of other hospitals delivering trauma care for less complex cases (trauma units (TUs)). These TUs will also be identified within each SHA in collaboration with Primary Care Trusts (PCTs)/Primary Care Consortiums, and assigned the management of less complex injuries, providing mostly limited and selected trauma care. At a third level, local hospitals will continue to provide existing emergency department services for minor injuries. The relationship and collaboration of the different healthcare facilities of different levels within the same regional network (MTC and TUs) is assured from the first phase of designation. Each SHA is responsible for facilitating and coordinating the establishment of robust communication pathways, and all stakeholders need to appreciate the vital role of collaboration at all levels. Existing pathways between hospitals and health services under the current secondary/tertiary referral system may also be utilised as scaffolds for the future RTN structure. Major threats of failure of this proposed RTN structure include congestion of the MTCs and segmentation of patient care between the MTC, peripheral units and rehabilitation [31].

The existing practice of consultant-led resuscitation available mostly in working hours will change to a 24-h consultant presence 7 days a week in all MTCs. Equally, consultant-led services will be available in a short timeframe post admission for all relevant surgical specialties (Table 2). Obvious requirements for the appointment of Accident and Emergency (A&E) consultants, trauma-oriented surgical specialists, as well as the recruitment of all necessary human resources and assets, are being addressed urgently in all SHAs and Trusts. Administrative and clinical personnel focused specifically on the care of the injured patients appear to be a major shift in health-related human resources over the last few years. To that end, institutions that have been already functioning as tertiary referral centres have the highest chance of becoming MTCs or TUs, making the selection process by the SHAs less difficult.

Trauma-related technical skills, training and professional education has been mostly provided by medical colleges, including courses for surgeons and other clinicians via the Advanced Trauma Life Support (ATLS) course, and for paramedics via the Pre-Hospital Trauma Life Support (PHTLS) course, which have withstood the test of time and have proven invaluable [31]. Further exposure and educational opportunities are currently available and will be explored, mostly related to the experience gained from the military and its advanced practices [55-58]. Education, however, should expand to include development of non-technical skills (leadership, communication, situation awareness, teamwork) [59,60], as well as addressing the necessity for increased public awareness and prevention measures. Primary Care Trusts and the public must be active participants in the RTN. They need to be informed of the ways to access it, collaborate with relevant individuals and professional groups within it, and how to interact and participate in its preservation and improvement. The London and East Midlands SHAs have incorporated these aspects of public training and interaction [32,61] and their experience will be useful in the development of further networks across the country.

Specific triage protocols have been introduced in London to support decisions regarding which patients should be taken to the MTC on the basis of an assessment of patient physiology (vital signs, consciousness), anatomy of apparent injuries, mechanism of injury, and individual patient factors (for example, age extremes, pregnancy, obesity) [32]. An anticipated overtriage of patients towards the MTCs is expected, but with time and with all the appropriate measures in place it is envisaged that this concern will be addressed. The role of the regional Ambulance Services in the specific design and implementation of these screening tools is essential. Each SHA is expected to involve them, as well as the rest of the stakeholders, taking into consideration local parameters in each region.

Transportation of trauma cases to the appropriate hospital in the network will become effective via the standard ambulance land and air services. Modern screening protocols, coordination by senior clinicians, and rationalisation of the geographic distribution of the TUs and the MTC(s) in each region is expected to secure the delivery of the appropriate level of care at the appropriate time for every injured patient. An acceptable transportation time within 30-45 min is expected to have minimal adverse effect on the outcome of trauma cases [41], as long as ambulance paramedic personnel are well trained and equipped, and the receiving hospital is of the appropriated level for the patient's needs.

As identified mostly from the interaction with public and the PCTs from the London and East Midlands pilot work, the support of families and carers, availability of counselling services, involvement of the above in the decisions about continuum of care and choice of TU to be transferred to post MTC hospitalisation, are of great importance. Patients should be taken to the best-equipped hospital for their particular injury, even if this means a slightly longer journey. MTCs must also fulfil minimum requirements of easy access in terms of public transportation, parking, accommodation and childcare. Each RTN must optimise all these parameters for the patients and their carers, making the whole experience of trauma care less stressful [31,32,37,39,61].

Robust communication systems and pathways are another of the major pillars of the designed RTNs. Direct access lines and around the clock, readily-available contacts will be accessible to all relevant stakeholders. It will ensure the continuity of care between MTC, TUs, rehabilitation units and general practitioner (GP) consortiums.

The current status of underprovision of rehabilitation services, especially to major trauma patients, is expected to be reversed by prompt engagement of these high-demand cases with complex and specialist rehabilitation practitioners. A more centralised rehabilitation framework with synchronisation of all the available resources is desirable, which would allow a more self-directed therapy based on the individual demands of the patient [36]. Rehabilitation will start intensively from the acute phase, and continue uninterrupted following patient repatriation. Inevitably, redistribution of the available human and material resources, as well as additional specialists and infrastructure facilities, will be required. At a local level the early engagement of all stakeholders from all different trauma-related groups is initiated. The identification of the currently available capacity across the span of each region and the prerequisites for each individual health facility to function in its regional network is also underway. Specific workgroups in each SHA have been formed over the last year to ascertain the methodology and keep to the tight timelines. Effective leadership will be needed to drive the rehabilitation of major trauma patients within and outside the hospital setting from the early stages of their admission. Patients will be given a prescription for rehabilitation and specialised personnel will be involved from the very early stages, coordinating the overall rehabilitation course of each patient, preventing delays and optimising the final outcome. Directories of available rehabilitation services and contacts across each RTN will be developed to accommodate this increased workload [62].

Data currently collected by the Trauma Audit and Research Network (TARN) has matured over the last two decades and is expected to expand and incorporate all trauma-care-providing hospitals. Specific steps and plans have been made to erase existing financial or structural arguments for non-participation in the TARN family. In the fast-approaching new era, all hospitals participating in each trauma network will by default contribute to the TARN data collection, and are expected to work together sharing data collection assets and personnel. The new tariff for major trauma being developed by the DoH is expected to include an uplift to cover the cost of TARN membership [62]. The currently-collected information includes all the key observations, investigations and interventions through the patient's pathway, details on the injury mechanism, and injury descriptions that allow the assignment of injury scores and outcome on discharge from about 90% of England's trauma-receiving hospitals [63]. The recorded data include all patients that sustained a traumatic injury and whose length of stay was 72 h or more and/or all trauma-related deaths, admissions to intensive care units (ICUs)/high-dependency units (HDUs), and/or transfers from another hospital for specialist care. The expectation is to achieve 100% of compliance and submission of data from all hospitals participating in the RTN by the end of 2011 [63]. In parallel, the DoH and the TARN are expected to develop measures of outcome additional to the evidence collected from TARN that will cover the entire pathway of the injured patients from prehospital care to rehabilitation. All this evidence will be used to benchmark performance between SHAs, Trusts, MTCs, TUs, Ambulance Trusts, and rehabilitation units at a regional and national level. The role of the National Institute of Health and Clinical Excellence (NICE) in accordance with the DoH will be to develop the long-anticipated standards of trauma care, monitor their effectiveness, and propose changes in due time. The function of the MTCs and TUs is expected to be revalidated and monitored based on the TARN evidence, and key performance indexes in comparison to their peers and international standards regarding mortality, length of hospital stay, patient-reported outcome measures and trauma-specific outcome measures that will be introduced [64,65].

The role of the trauma network in trauma-specific clinical research, education and prevention will be another basic pillar and standard item of the network's agenda [66]. Each MTC will be linked with local Comprehensive Local Research Network (CLRN) specialty groups and higher education institutions, funding bodies and National Institute of Health Research (NIHR) network, contributing substantially to the development and realisation of trauma-related research at a national level [62].

The financial aspects of this effort have also been evaluated and significant steps forward have been introduced by the National Clinical Trauma Director [65]. The calculated economic cost of the RTN implementation nationally is expected to be cost neutral, and progressively will evolve into a cost reduction system [65]. According to the Trauma Char on current figures the costs of implementation will range from £1.4 million to £2 million per MTC [67]. Their variation is attributed to the differences between the centres' present state of providing specialist services and their existing infrastructure. Further cost-effectiveness evidence based on the available data on modelling and monitoring the trauma-related burden led to the conclusion that a regional trauma system in England will be cost effective if the initial investment for the RTN full implementation nationally is less than £60-70 million [68].

Additional expenditure will be needed to cover the additional transfer costs of trauma patients (initial bypass protocols, repatriation transfers), as well as for the early ample involvement of the rehabilitation services. Evidence from the international literature suggests that with basic drives the reduction of hospital and ICU stay after the first years of the system's maturation (a period of 2-3 years) a positive income shift should occur. The increased direct medical cost due to the anticipated effect on the survivorship of the trauma population should also be evaluated, calculated and claimed in order to have a balanced economic future. A reform of the application of existing HRG-4 based on payment by results (PbR) tariffs in the case of injuries has been announced and is expected to allow the rationalisation of the hospitals reimbursement from April 2011. Crossmatching the diagnosis scores, based on the World Health Organization *International Classification of Diseases*, tenth edition (ICD-10), with the intervention/procedure complexity will produce a more realistic model of coding and refunding. With the centralisation of major trauma services, foreseeable improvements on length of hospital stay and outcome, in combination with the use of appropriate coding models, the RTNs will be able to sustain the new trauma services, compensating and justifying all necessary investments. In addition, critical care tariffs will be mandated as bed-day rates from April 2011, diminishing the weaknesses of the previously block contracts and allowing the expansion of ICU capacity at MTCs if and when required. From a wider perspective, using estimates from the Department of Transport [69] regarding the direct and indirect costs of each road traffic accident (RTA) victim (£1.64 million per fatality and £185,000 per serious injury in 2007 values), and the estimated reduction in such fatalities according to the recent prediction of the National Audit Office (NAO) of 600 lives per year [39], the savings could well reach the sum of £879 million each year in England.

### Future challenges

Establishment of specialist RTN services and adoption of a robust clinical governance programme can achieve significant improvements in the process of care and outcomes from severe injury [33]. The proposed inclusive system will incorporate the MTCs, local TUs, and ambulance and rehabilitation services (hospital and community) into one network directory. This seamless, linked system will optimise patient outcomes and concurrently reduce lengths of hospital stay, rationalise the use of intensive care beds, reduce disability and improve occupational return, with an overall improvement in patient experience.

There are a number of anticipated challenges that each of the components of the RTN will face in the near future. Further, it is expected that numerous other problems will arise that local and national leaders and stakeholders will have to overcome in this endeavour.

The biggest challenge facing the RTN is to constructively utilise the diversity, complexity and uniqueness of individuals and organisations in a finely tuned system for prevention of injury and provision of high quality of care for all injured patients.

In addition, financial limitations are also a major obstacle, especially if the approach to the problem is confined to a short period of time. However, in the mid to long term there is no reason that restructuring of our failing trauma services will not be proven cost effective at local, regional and national levels [38].

Another critical point with regard to the excellent paradigm of the London RTNs, the pioneers of this national trauma reformation, is that certain lessons can be learnt even though a complete replication of their model in other regions of the UK would be impractical. The dense urban character of the capital is not met even in other large urban areas of the country. Further, a significant variance can be anticipated in the sheer numbers of major trauma cases that each MTC will treat annually. The determination of whether a lower threshold of 250 cases annually [41], in order to maintain the necessary levels of readiness and expertise, is adequate or achievable, can only be verified over time.

It is clear from the international experience of regionalisation of trauma services that safe conclusions of its effectiveness at a larger or a local scale can be evaluated after the first 2-5 years of implementation [49,50,70]. This transitional period for the RTN system may prove to be demanding and may raise significant dilemmas and questions as to the suitability of the project for the whole of the UK, or for some of the peripheral regions. For this reason, the role of each SHA, PCT and local Trust is essential in fine tuning the general idea to accommodate the specific needs and characteristics of each region. Patience and persistence in this endeavour are of paramount importance to this mid-term to long-term investment in human resources, assets, and efforts. The foreseeable initial improvements are expected to be related to complex trauma disability rates, and secondarily to mortality.

The most unexplored component of RTN implementation is related to rehabilitation services. The existing capacities of these services in the hospital as well as in the community setting are not recorded, nor addressed in the pilot model of London, at least in comparison to the other essential aspects of RTNs. Significant variance is expected to exist between different SHAs and regions, whilst the financial aspects of their upgrading also remain unknown. The significance of rehabilitation services is crucial as its failure will lead to the early congestion and suffocation of MTCs, and will undermine the expected improvements in disability rates of the injured patients during the first years of the RTN system.

Present data recording and quality assurance are based solely on the TARN database, which under-represents the clinical reality as it collects data from the majority of but not all trauma-covering hospitals, and is mostly based around fatalities in admitted patients. Nevertheless, the quality of this database, and the support it receives from the national trauma leaders, offers a strong starting point. The incorporation of data from ambulance services and rehabilitation units is expected to produce an amalgamation of collected evidence that will surpass any previous audit attempt. Obvious organisational and administrative demands for such a task should not be underestimated, and remain unspecified in the existing RTN national plan.

## Conclusions

The reform of trauma care in England, especially the care of severely injured patients, has already started, with strong clinical leadership being embraced as the way forward. There is no doubt that the trauma-related healthcare community is finally, after many years, under the spotlight. However, one may argue that the timing may not be perfect due to the current unprecedented economic climate. Moreover, the expectations are high and time is pressing. While for the first time ever a genuine political will and support exists, the changes required to maintain the momentum for the implementation of the RTN need to be marshalled against arguments, myths and perceptions from the past. Such an approach may reverse the disinterested attitudes of many, and gradually produce a cultural shift in the public, clinicians and policymakers over time. Only then can a successful implementation of the RTN concept be expected and the long-aspired optimisation of the care of traumatised patients in England attained.

## Competing interests

The authors declare that they have no competing interests.

## Authors' contributions

NKK contributed to the design, acquisition of relative data, drafting of the manuscript. PVG contributed to the conception, design, and final drafting of the manuscript. Both authors read and approved the final manuscript.

## Pre-publication history

The pre-publication history for this paper can be accessed here:

http://www.biomedcentral.com/1741-7015/9/121/prepub
